# Supplementary material for: Lupus enhancer risk variant causes dysregulation of IRF8 through cooperative lncRNA and DNA methylation machinery
Source: Nat Commun. 2022 Apr 6;13:1855. doi: 10.1038/s41467-022-29514-y (PMC8987079; doi:10.1038/s41467-022-29514-y)
Supplement: Supplementary file 1 — Supplementary Information [file 41467_2022_29514_MOESM1_ESM.pdf]

CRISPRa screen on a genetic risk locus shared by multiple autoimmune diseases identifies a dysfunctional enhancer that affects *IRF8* expression through cooperative lncRNA and DNA methylation machinery

Supplementary information

Supplementary Figures

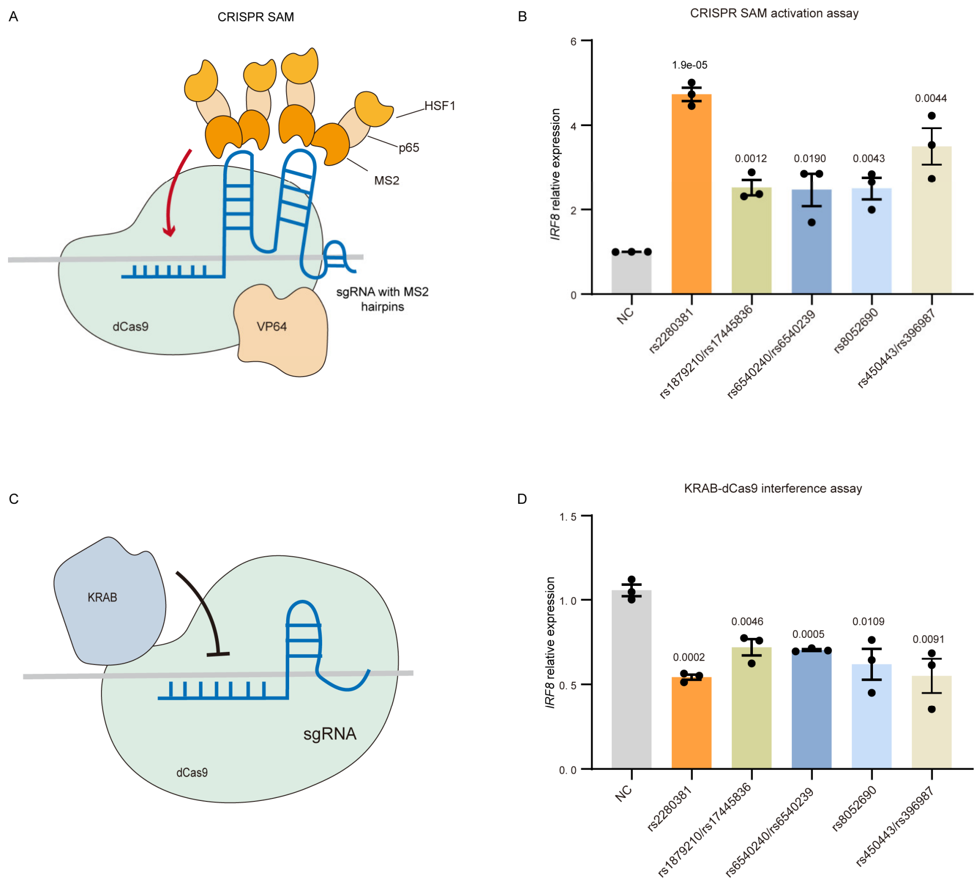

Supplementary Fig. 1 Related to Fig. 1: CRISPR screen identifies the functional genetic variants' regions regulating *IRF8* expression. (**A**) The components of CRISPR SAM activation system. **(B)** RT-qPCR analysis of *IRF8* expression in the CRISPR SAM activation experiment (n = 3, biologically independent experiments). **(C)** The components of KRAB-dCas9 system. (**D**) RT-qPCR analysis of *IRF8* expression in the KRAB-dCas9 interference experiment (n = 3, biologically independent experiments). Data are represented as mean ± SEM, and *P*-values are calculated using an unpaired two-tailed Student's t-test.

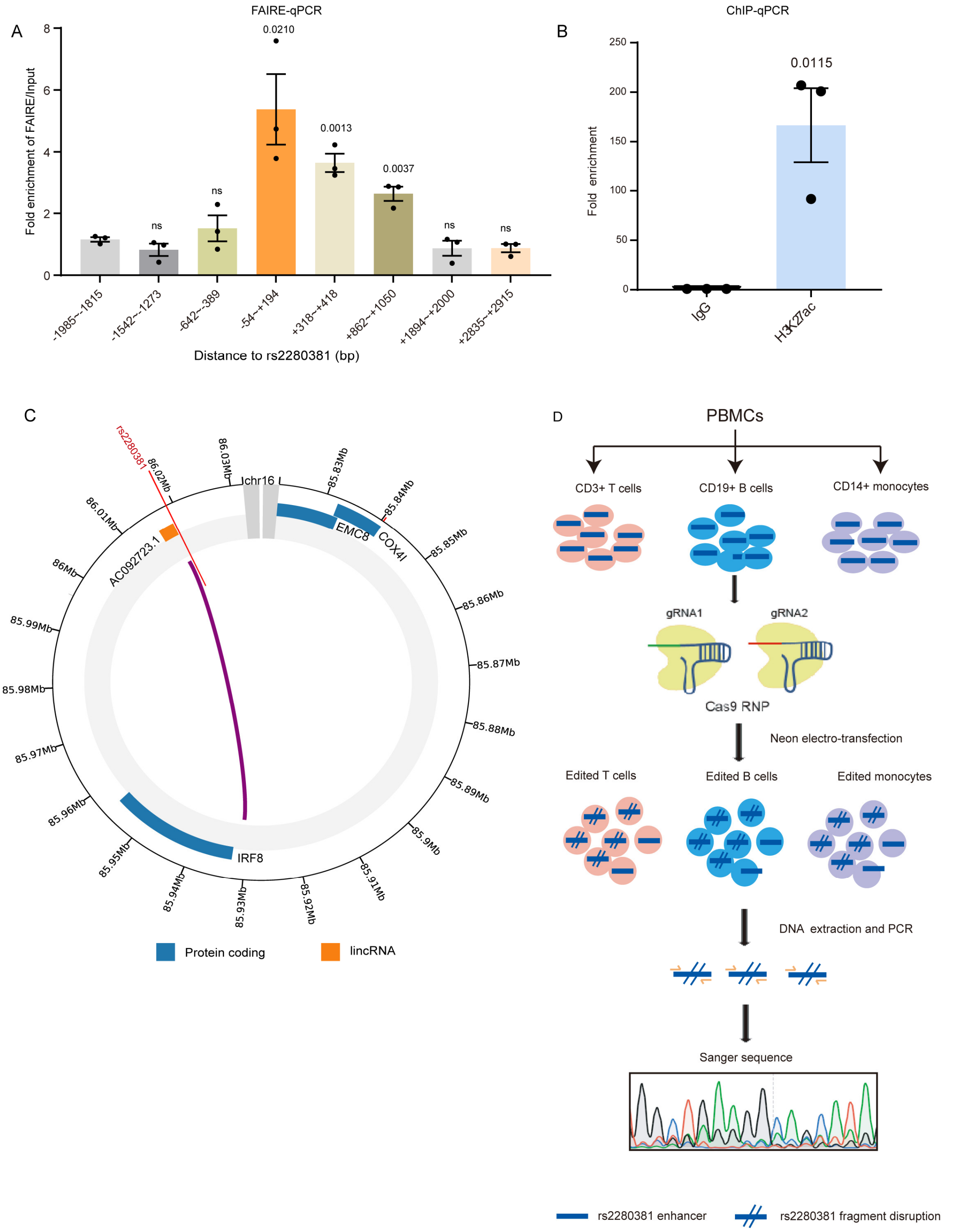

Supplementary Fig. 2 Related to Fig. 2: (A) FAIRE-qPCR analysis of chromatin accessibility within the rs2280381-containing region (n =3, biologically independent experiments). **(B)** Analysis of H3K27ac signal within the rs2280381-containing region in U-937 cells by ChIP-qPCR (n =3, biologically independent experiments). (**C**) The circos plot visualizing significant *cis* interactions detected by 4C-seq in primary

monocytes. The black circle represents the topologically associated domain (TAD) domain around rs2280381. For each gene in this region, coding genes are depicted (without the exon/intron details) in blue and lncRNAs are depicted in orange. The orange line indicates the position of rs2280381, the purple line indicates the connection between the *IRF8* promoter and rs2280381. **(D)** Flow scheme of CRISPR-mediated fragment disruption in isolated primary cells. Data are represented as mean ± SEM, and *P*-values are calculated using an unpaired two-tailed Student's t-test. ns: not significant.

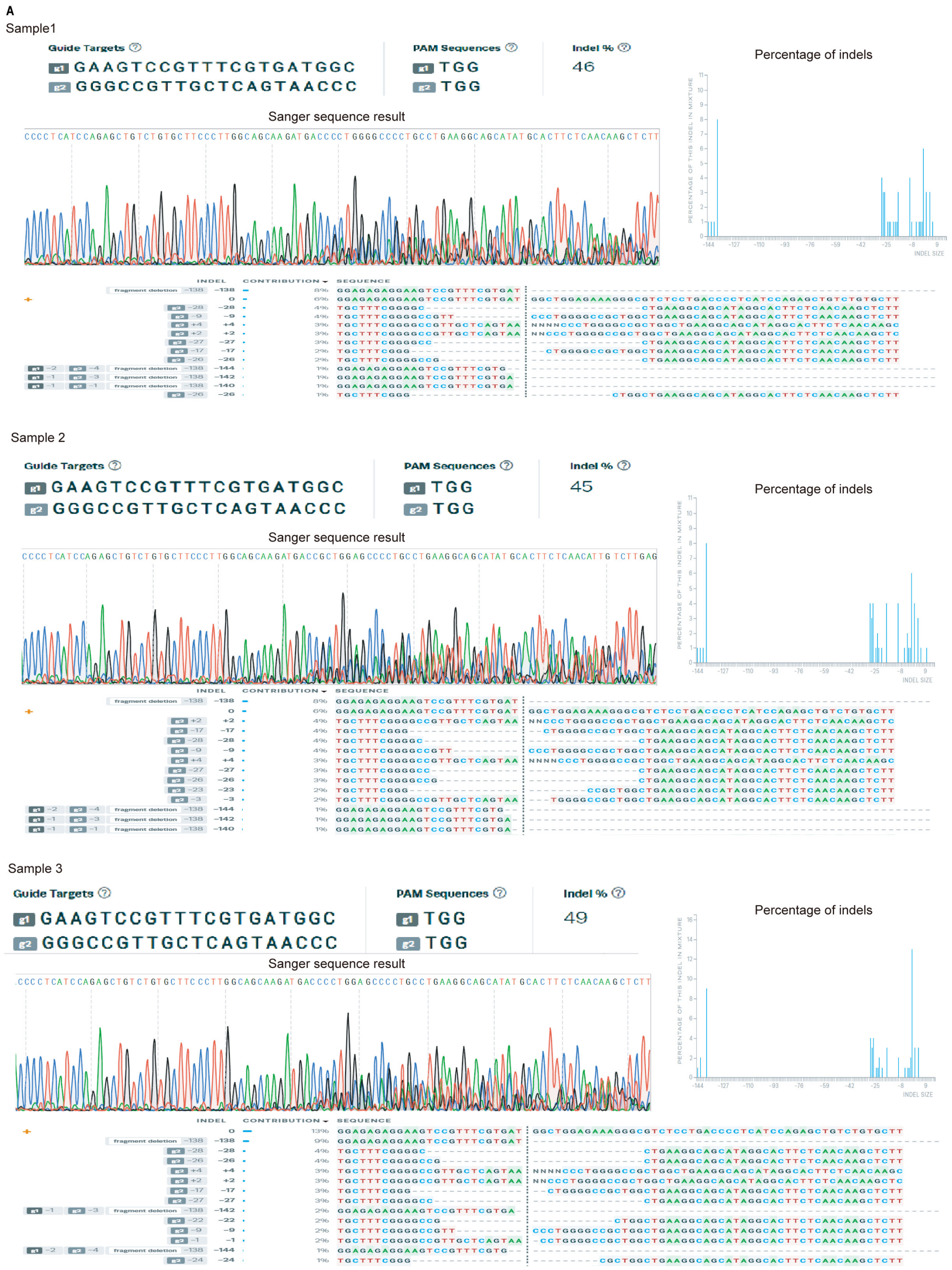

Supplementary Fig. 3 Related to Fig. 2: ICE analysis the of the indel frequency at rs2280381 locus in the edited human CD3+ T cells.

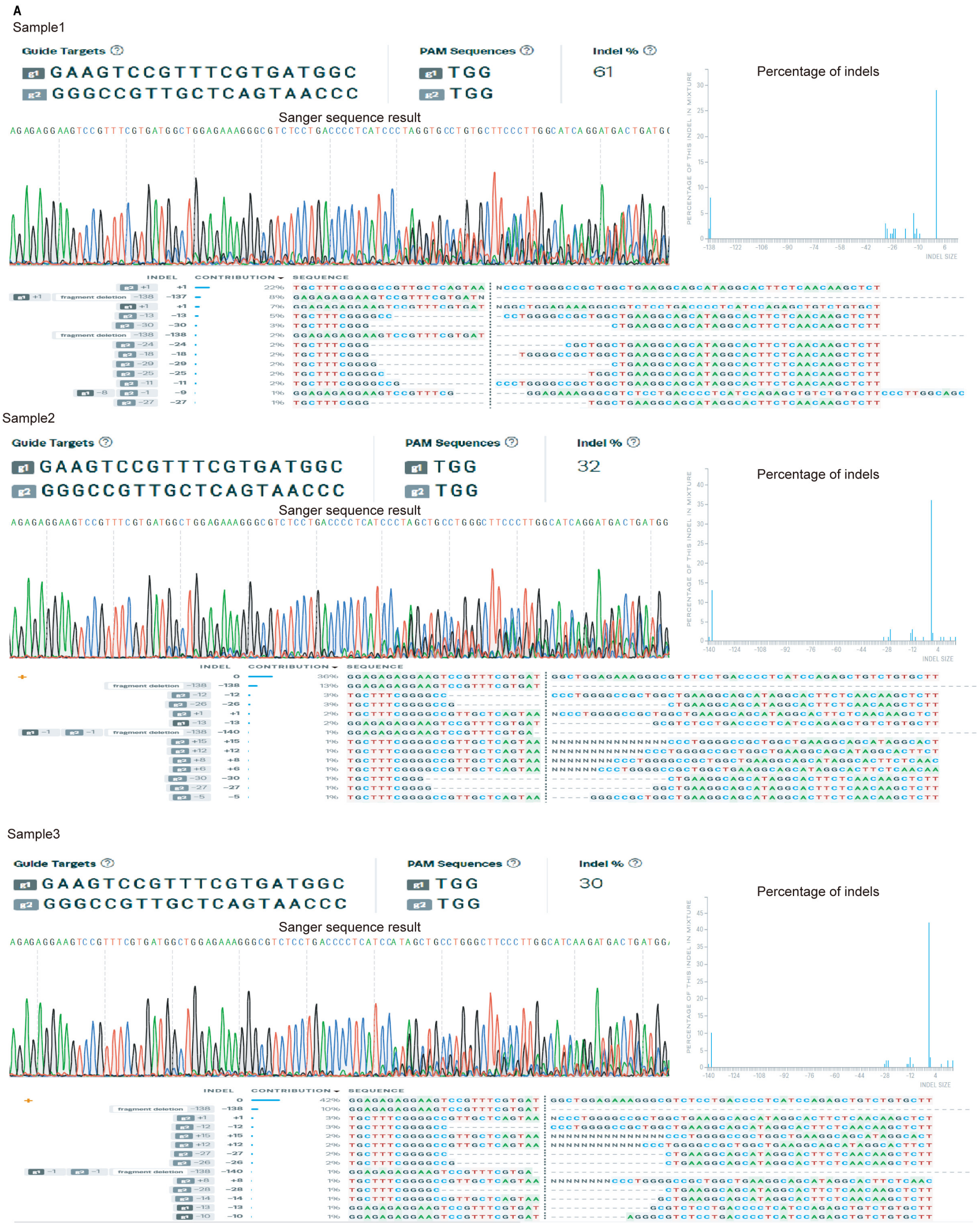

Supplementary Fig. 4 Related to Fig. 2: ICE analysis the of the indel frequency at rs2280381 locus in the edited human CD19+ B cells.

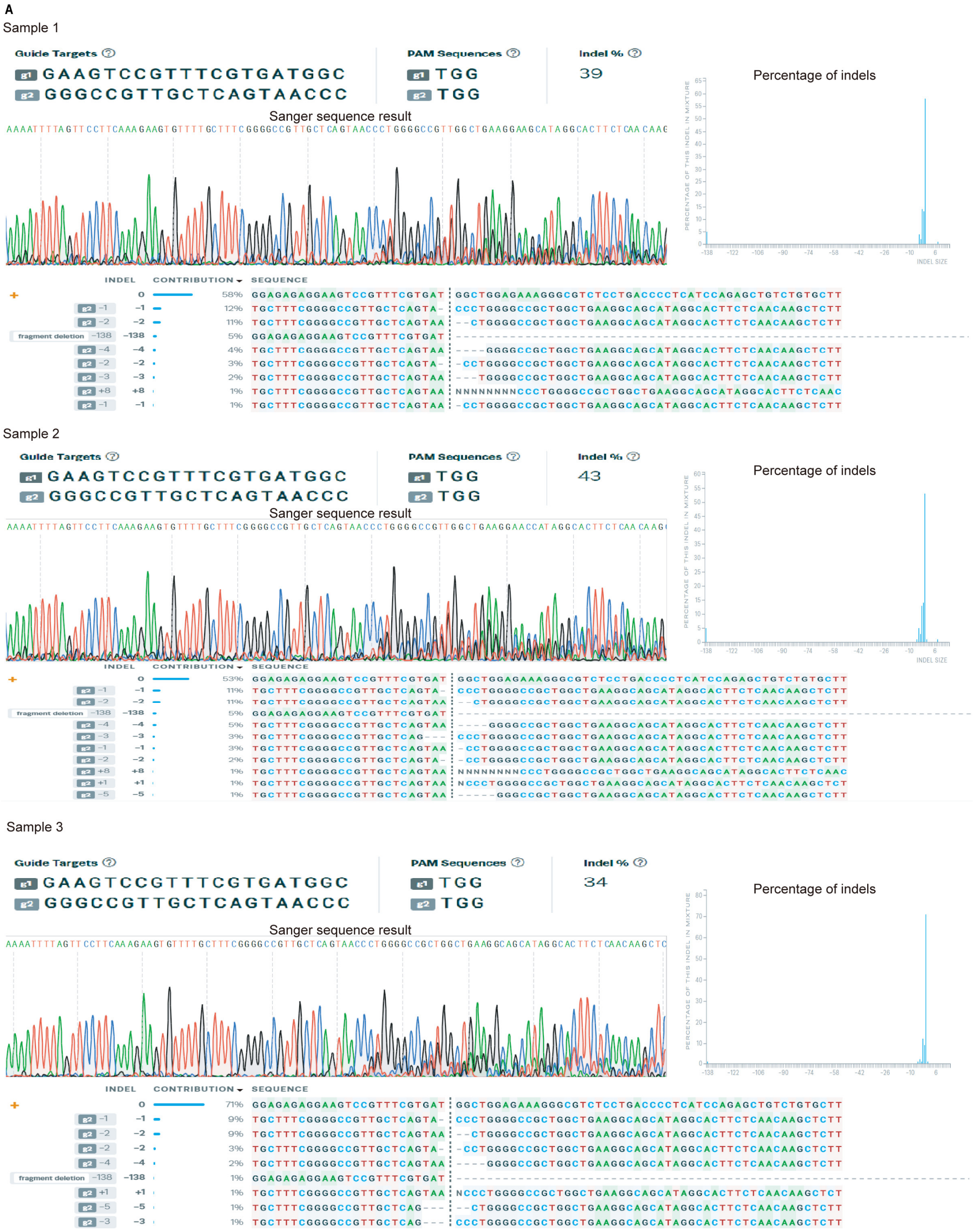

Supplementary Fig. 5 Related to Fig. 2: ICE analysis the of the indel frequency at rs2280381 locus in the edited human CD14+ monocytes.

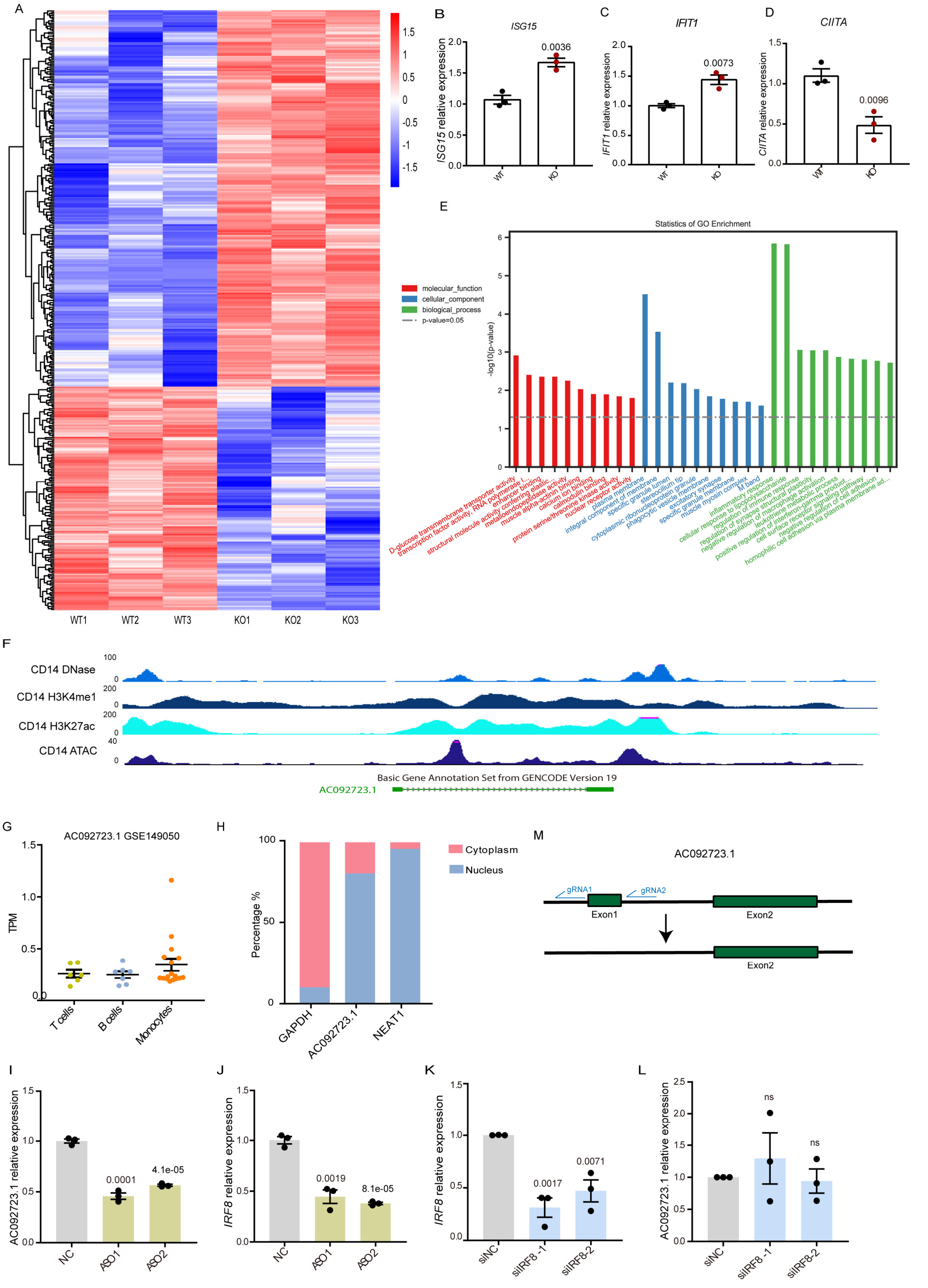

Supplementary Fig. 6 Related to Fig. 3: (A) The heatmap of differentially expressed genes regulated by the rs2280381-containing region as measured by RNA-seq (log2 fold change ≥1 and FDR<0.05). The red and blue colors in the heat map depict higher and lower gene expression, respectively. The color intensity indicates the magnitude of the expression differences. (**B-D**) RT-qPCR analysis of *ISG15*, *IFIT1* and *CIITA*

expression in WT and rs2280381 KO clones (n = 3, biologically independent samples). WT: rs2280381 wildtype, KO: 138 bp fragment containing the rs2280381 deletion. **(E)** Top 10 GO categories associated with differentially expressed genes. Categories were ranked by -log10 (*P* value). **(F)** AC092723.1 is located in an enhancer region with strong H3K4me1, H3K27ac, DNase and ATAC signals. **(G)** AC092723.1 expression in different human immune cell subpopulations as measured by RNA sequencing from public database. **(H)** RT-qPCR analysis of AC092723.1 abundance in nuclear and cytoplasmic fractions of U-937 cells. *GAPDH*, cytoplasmic marker. NEAT1, nuclear marker, (n = 3, biologically independent experiments). (**I-J**) RT-qPCR analysis of AC092723.1 and *IRF8* expression with or without AC092723.1 knockdown in U-937 cells (n = 3, biologically independent experiments). (**K-L**) RT-qPCR analysis of AC092723.1 and *IRF8* expression with or without *IRF8* knockdown in U-937 cells (n = 3, biologically independent experiments). **(M)** Deletion of the exon1 fragment of AC092723.1 using CRISPR-Cas9. Data are represented as mean ± SEM, and *P*-values are calculated using an unpaired two-tailed Student's t-test. ns: not significant.

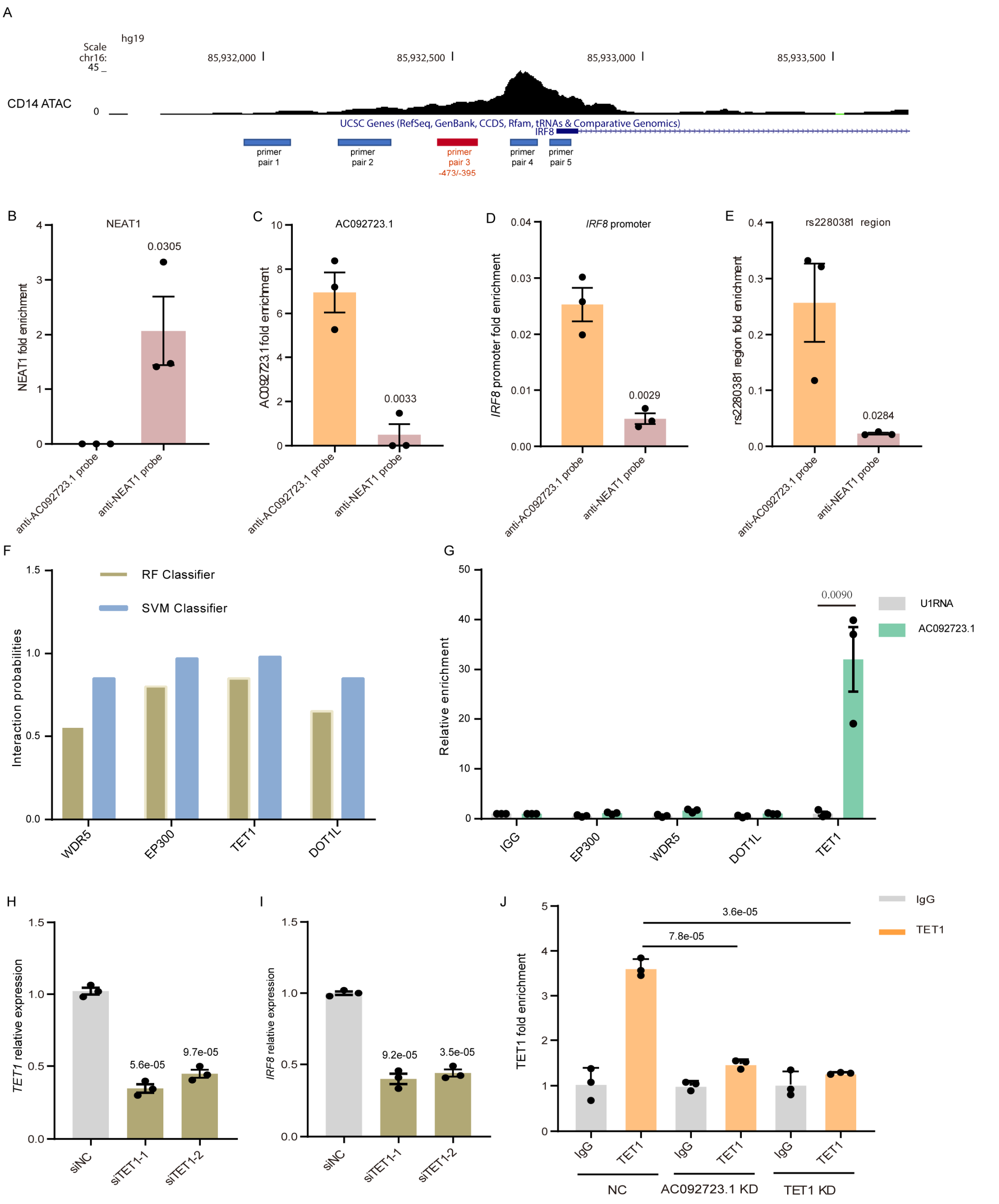

Supplementary Fig. 7 Related to Fig. 4: (A) The position of ChIRP primers in the *IRF8* promoter region. **(B-C)** NEAT1 RNA and AC092723.1 RNA are enriched explicitly with anti-NEAT1 probes (B) and anti-AC092723.1 probes (C) in ChIRP assay in U-937 cells, respectively (n = 3, biologically independent experiments). **(D-E)** AC092723.1 interacts with the *IRF8* promoter region (D) and rs2280381-containing region (E) in U-937 cells (n = 3, biologically independent experiments). **(F)**

Bioinformatic analysis of AC092723.1 interacted chromatin modifiers using online tool RPISeq based on random forest (RF) or support vector machine (SVM) models. Interaction probabilities generated by RPISeq range from 0 to 1, predictions with probabilities > 0.5 indicating that the corresponding RNA and protein are likely to interact. (**G**) RIP-qPCR analysis of the interaction between AC092723.1 and predicted binding chromatin modifiers in U-937 cells (n = 3, biologically independent experiments). (**H-I**) RT-qPCR analysis of *TET1* (H) and *IRF8* (I) expression in U-937 cells after knockdown of *TET1* by siRNA (n = 3, biologically independent experiments). (**J**) ChIP-qPCR analysis of the binding efficiency of TET1 to the *IRF8* promoter with or without AC092723.1 knockdown in U-937 cells (n = 3, biologically independent experiments). Data are represented as mean ± SEM, and *P*-values are calculated using an unpaired two-tailed Student's t-test.

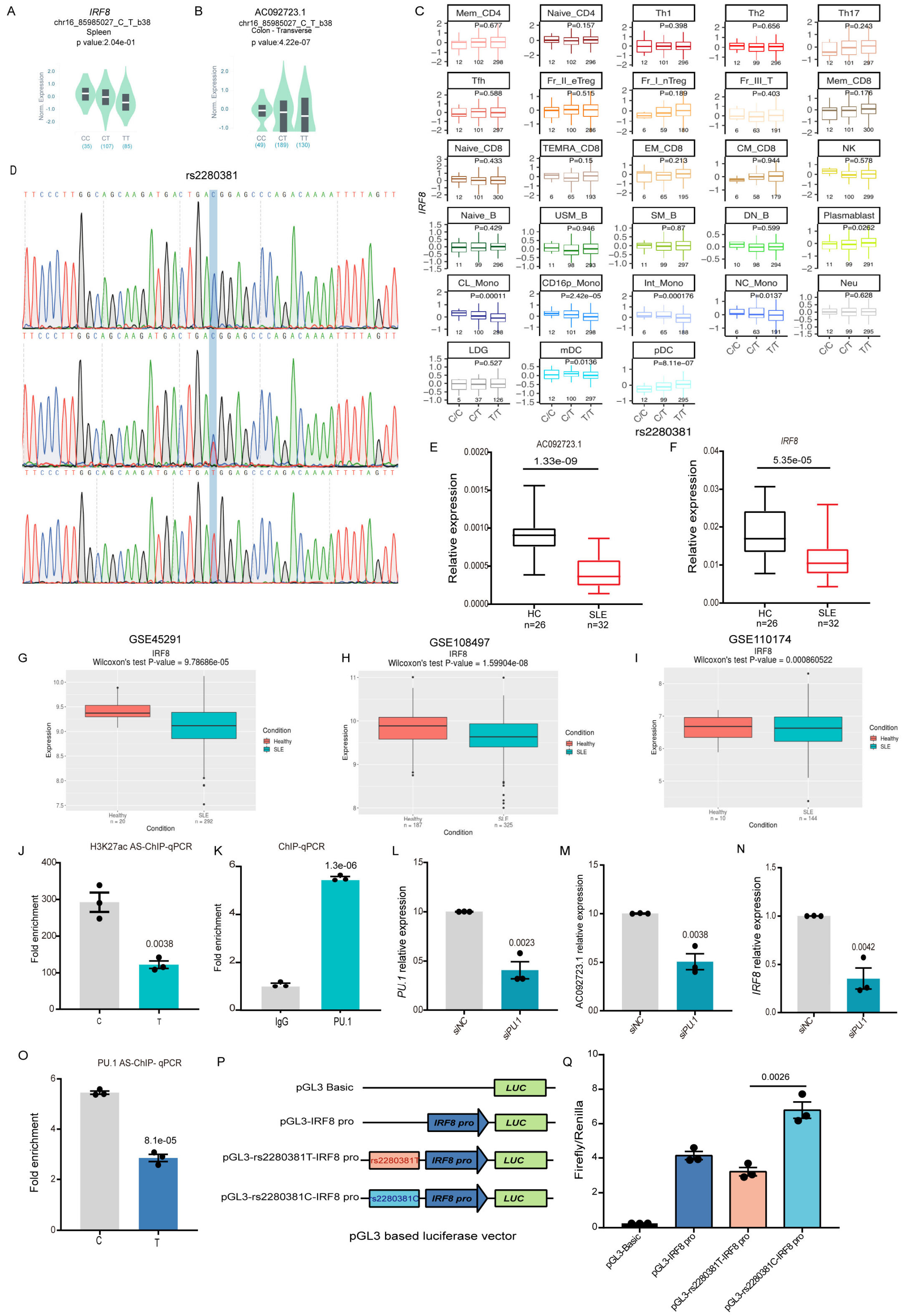

Supplementary Fig. 8 Related to Fig. 5: (A-B) GTEx data analysis of the eQTL between *IRF8* or AC092723.1 and rs2280381 allele. **(C)** The box plot for eQTL result between *IRF8* and rs2280381 allele in different immune cell subpopulations from ImmuNexUT database. **(D)** Genotype of clones was identified by Sanger Sequence. **(E-**

F) The box plot for AC092723.1 and *IRF8* expression in SLE patients examined by RT-qPCR (n = 26 biologically independent samples for HC, n = 32 biologically independent samples for SLE). **(G-I)** Public RNA profiling data indicates the low expression of *IRF8* in SLE patients in box plot format. (**J**) rs2280381 non-risk C allele enriched more H3K27ac marks than risk T allele, as determined by AS-ChIP-qPCR in the rs2280381 heterozygous U-937 cell clone (n = 3, biologically independent experiments). (**K**) Relative enrichment of PU.1 binding to the rs2280381-containing region, as measured by ChIP-qPCR in U-937 cells (n = 3, biologically independent experiments). **(L-N)** Relative expression of *PU.1* (L), AC092723.1 (M) and *IRF8* (N) after PU.1 siRNA-mediated knockdown, as measured by RT-qPCR in U-937 cells (n = 3, biologically independent experiments). (**O**) PU.1 prefers binding to the rs2280381 C non-risk allele, as determined by AS-ChIP-qPCR in the rs2280381 heterozygous U-937 cell clone (n = 3, biologically independent experiments). (**P**) Plasmid schematic of *IRF8* promoter pGL3 and rs2280381 enhancer-*IRF8* promoter pGL3 luciferase report vector. (**Q**) Luciferase reporter assay with PU.1 over-expression in HEK-293T cells (n = 3, biologically independent experiments). Box plot: centre line, median; box limits, the interquartile range (IQR); and whiskers, the maximum and minimum values no further than 1.5 × IQR from the hinge. Data are represented as mean ± SEM and *P*-values are calculated with two-sided tests for linear regression coefficients without adjustment by multiple comparisons (A-C) or an unpaired two-tailed Student's t-test (E-F, J-O, Q) or a two-sided Wilcoxon signed-rank test (G-I).

Supplementary Tables

Supplementary Table 1

RT-qPCR primers used in this study

RT-qPCR Primer	Sequence (5'-3')
GAPDH-F	GGAGCGAGATCCCTCCAAAAT
GAPDH-R	GGCTGTTGTCATACTTCTCATGG
IRF8-F	ATCAAAAGGAGCCCTTCCCC
IRF8-R	GGGAGAATGCTGAATGGTGC

AC092723.1-F	TGTGGTTTCAATGGAGCCCT
AC092723.1-R	TGTGCTACACAGGAAACGCT
U1RNA-F	CCATGATCACGAAGGTGGTTT
U1RNA-R	ATGCAGTCGAGTTTCCCACAT
TET1-F	TAGCCCAACTCTCTCAGGCT
TET1-R	GCACTGGCATAGTCACCACT
PU.1-F	CCTGAGGGGCTCTGCATTG
PU.1-R	GAAGTTGTTCTCGGCGAAGC
ISG15-F	CGCAGATCACCCAGAAGATCG
ISG15-R	TTCGTCGCATTTGTCCACCA
IFIT1-F	AGAAGCAGGCAATCACAGAAAA
IFIT1-R	CTGAAACCGACCATAGTGGAAAT
CIITA-F	CCTGGAGCTTCTTAACAGCGA
CIITA-R	TGTGTCGGGTTCTGAGTAGAG

Supplementary Table 2

4C-seq primer used in this study

Name	Sequence (5'-3')
P5-381-MN-F	GAAACCACAAGTCGGATC
P7-381-MN-R	AGAACTGAATGCCGAAGC
P5-IRF8-CN-F	CCAGTTCCTGTAATGTAC
P7-IRF8-CN-R	AAGACCATTTTTCTGGCT

Supplementary Table 3

FAIRE-qPCR primer

Name	Forward (5'-3')	Reverse (5'-3')
-1985~-1815	ATAAGTGTGGAGCGCTTGCT	CCAGCTTCACCCATCACTGT
-1542~-1273	CGTTCAGGTGGGACTGAGAC	CAGAGCCAGACACCAGGAAG
-642~-389	CCGAGGATGGATCCGACTTG	CCAAGGTTCCAACAGCTTGC
-54~+194	TCCTGACCCCTCATCCAGAG	AAGACAGAGAGAACGCGGTG
+318~+418	GCTCCAAGCATGGTCAGACT	CTCAGGGTATTTCCTGGGCG
+862~+1050	CGTAGTGACACCAACCACGA	GCGATCCCCCATTTCACAGA
+1894~+2000	TGCCTCCGTAAGTCCAACAC	GTTTCTCAGCTGGGAGGGAC
+2835~+2915	CCTGTTGGGCTGCTGGATAA	GGCTCATATTCTCGGGAGGC

Supplementary Table 4

Allele-specific qPCR primer

Allele specific primer	Sequence (5'-3')
381-AS-C-F	GGCAGCAAGATGACTGAC

381-AS-T-F	GGCAGCAAGATGACTGAT
381-AS-R	CCAGGCGTGTACTCAAGA

Supplementary Table 5

CHIRP probe sequences used in this study

probe	Sequence (5'-3')
AC092723.1-1	AACCACAAGTCGGATCCATC
AC092723.1-2	CTCACGGAGAGGGTAAGTGA
AC092723.1-3	TTTGGGAGAAGAGTCTGTGC
AC092723.1-4	GAATTGGGACATTGGTGTGG
AC092723.1-5	GCATTCAGTTCTACTCTTTT
AC092723.1-6	CGTTTCCAAGGTTCCAACAG
AC092723.1-7	AATTACAGGCAGAGGCAAGA
AC092723.1-8	ACCTACTTTCATCCTTACAA
Neat1-1	GCAAAGGTACATGGATTCTG
Neat1-2	ATTTTAGGTGATAGTTTCCC
Neat1-3	CATGTAGTAAAGGCACCTCG
Neat1-4	CCATTGGTATTACTTTACCA
Neat1-5	GGTTAAGAACTGAACTACCA
Neat1-6	TTGTTTGCATCATCCCCAAG

Supplementary Table 6

CHIRP experiment detection primer

CHIRP detection primer	Sequence (5'-3')
Neat1-F	AGCTGCGTCTATTGAATTGGTAAAGTAA
Neat1-R	GACAGAAAGATCCCAACGATAAAAATAA
RT-AC092723.1-F	TGTGGTTTCAATGGAGCCCT
RT-AC092723.1-R	TGTGCTACACAGGAAACGCT
rs2280381-F	GCCCCGAAAGCAAAACACTT
rs2280381-R	CTCCTGACCCCTCATCCAGA
IRF8-pro-1F	ATGCCAGGAGTTGACCTGTG
IRF8-pro-1R	GCAGAGCATGGTCCCAGATT
IRF8-pro-2F	TAAAAGCCAATCGCGACCCT
IRF8-pro-2R	TGGGTGGGCGTTAAGATGTC
IRF8-pro-3F	ATATCCAGCGCTCGTGAAGG
IRF8-pro-3R	CCTGCGGCCCATTAATCAGA
IRF8-pro-4F	GCCTAGCACCTTCGATGCTC
IRF8-pro-4R	GTCGTTTACAGACCGTCCCG

Supplementary Table 7

ChIP-qPCR primer

Name	Sequence (5'-3')
381CHIP-F	CTCCTGACCCCTCATCCAGA
381CHIP-R	GCCCCGAAAGCAAAACACTT

Supplementary Table 8

CRISPRa screen gRNAs

Target	gRNA1 (5'-3')	gRNA2 (5'-3')	gRNA3 (5'-3')
IRF8 promoter	TGGAGCGCGGCAGCAAGCGT	TTTTAAGGCCGCGCGACCGC	ACGGCGGCAGGTAGGCACAG
rs186249	CACAGGCACCACTAACGCTT	AGATTATGCCAGCAATCAAA	TGTGGAGGATCTGGTGTACA
rs2280381	CTTGGCAGCAAGATGACTGA	AGCACAGACAGCTCTGGATG	AAGGAACTAAAATTTTGTCT
rs8052690	GTGAGCCATCGACCTCACAG	GGTGCTCTGGACTGATGAAA	GTACTCTGCCCCAAGCACTT
rs8045825/ rs8059144	CAGAGCAGGCACTCGAGCGA	GCGTGTCCAGTCCACTCCCG	GCCTAGAAACTCAAACAGAG
rs4843867/ rs4843866	CTTGCTGAATGGGGACCAAG	CGATCACCAGGAGCCTCTCA	GTTAGCTCTCGCCTAATGAA
rs10711573 / rs11117432	GACAAACAAAACGTTGGAGG	TGGGCTTAATTTGGAAAACC	AAAAAAAACCCAACAGTGTG
rs4843865	CAACGACTCCTGCGATGACG	AGCAAGGAGATGGTTGATGG	CTCTTGGGTCCCTTCAAGCT
rs11347703	GCTCCGGGCACCCACGCTCT	GGGGTGGCACATGTCCTCAG	TCTGTGTGAGATTCCTATGG
rs450443/ rs396987	CACGTGTCCGTCACTTTATG	GCGCATCCGTGACACGACCT	TCCCTAAAATGTATAAAACC
rs731708	GAGGCAGGCAGTGCAACGGA	CGGGAGGAGGCTGACCTGGT	TGAGAGGGAGCCACGCAGAA
rs12149636	GCTCTTGCAAGATGTTGGGA	CTGTCTAGATAAACAGACTG	AGGTGGCATGGTATGTTCAC
rs11117431	AGATCTCGTCTCACTATACA	ACAAGAAAATGAAACTAGCC	CCTCCAACTCCTGGGCTCAA
rs6540241/ rs6540242/ rs6540243	GTGAAATGGGGGATCGCCGG	GGGGAAGGTAGGGGGCACAG	CTAGAAAATTGCAGAAAGAA
rs11117433	GGTGTCACTACGTATCCGAA	CAACCACGAGGTGGTCGGTG	CTGCTGGAGGTGCAAGACCA
rs1879210/ rs17445836	TGTCCCTAAACCAAGGCACG	GGGACAAGAGCCTCTGTCTC	TAGAAGGTGAGCCCGCAGCT
rs7203487	AAGTGTGGAGCGCTTGCTCT	GCTATGGTCTGCTTAGCACA	GTTCTTATCTCATAGTGTCT
rs6540240/ rs6540239	GGCTCCCACTCATCAGGACC	AAGACAGAGAGAACGCGGTG	TACCCGTAATATGAACGCAG
rs13330176	TTAGTTGTCTAATTTTCTCA	TGACTTTAACCTGCAGGTCA	TTTTGACACCGTGTCCGCCC
rs9674233	GCATCACAGAGCCGGTGTGA	TCAGTGATGGGTTCATGGCA	GTAACTGGTGGCGTTTCCAT
rs7202472	GAAGAGCCATGGACCATATG	GACACTGTGACTCTGAGTCA	ACTAACTGCCCCAAGCTCAG
rs9933582	GTGTCCCTCAAACTCAGGCC	ATAAGGCCTCCCGCAGTGCC	AATGAGAAGCGGGGGACAGA

rs9927316	GCCTCTCGTCTCCGTGTAAG	CTGACCGTTGAGACCTGGCG	GGCCTCTCGTGTCAGTGGGT
rs7193275	AATTCCTGTTTATTAAGGAC	ACAGACCAAGCATCTGTACC	AGCACAGACAGCTCTGGATG
rs9939427	CGGGGTGAATTGTGGTTGGG	GGACCTGTGAGCACCAGGGG	CTGATTCTGCCAAGTCCCCC

Supplementary Table 9

rs2280381 Prime editing gRNAs

rs2280381 Prime editing	Sequence (5'-3')
pegRNA	CTTGGCAGCAAGATGACTGA
RT template+PBS sequence	TCTGGGCTCCATCAGTCATCTTGCTGCC
nick gRNA	GCCCCAGGGTTACTGAGCAA

Supplementary Table 10

Knockout gRNAs used in this study

KO gRNA	Sequence (5'-3')	Target
gRNA1	GAAGTCCGTTTCGTGATGGC	rs2280381 KO
gRNA2	GGGCCGTTGCTCAGTAACCC	rs2280381 KO
gRNA3	AGATCTCGTCTCACTATACA	AC092723.1 exon1 KO
gRNA4	AGTCCATCACTGCTCCAGGA	AC092723.1 exon1 KO

Supplementary Table 11

ASO used in this study

AC092723.1 ASO name	Sequence (5'-3')
ASO nc	G*C*A*A*T*CGTGAGCAGA*C*C*C*C*T
ASO-1	C*C*T*C*A*CGGAGAGGGT*A*A*G*T*G
ASO-2	G*C*C*T*T*CATGCCCTCT*G*C*G*T*T

* Stand for Phosphorothioate modification

Supplementary Table 12

siRNA used in this study

siRNA Name	sense（5'-3'）	antisense（5'-3'）
si-IRF8-1	CCAUACAAAGUUUACCGAAUU	AAUUCGGUAAACUUUGUAUGG
si-IRF8-2	GCCCGCAUCAUGAUUAAAGAA	UUCUUUAAUCAUGAUGCGGGC
si-PU.1-1	GCAAGAAGAUGACCUACCA	UGGUAGGUCAUCUUCUUGC
si-PU.1-2	CAGAAGACCUGGUGCCCUA	UAGGGCACCAGGUCUUCUG
si-TET1-1	GCAGCUAAUGAAGGUCCAGAA	UUCUGGACCUUCAUUAGCUGC
si-TET1-2	CCUCCAGUCUUAAUAAGGUUA	UAACCUUAUUAAGACUGGAGG

Supplementary Table 13

DNA Pull down oligo used in this study

oligo	Sequence (5'-3')
381-C-F	TGTGCTTCCCTTGGCAGCAAGATGACTGACGGAGCCCAGACAAAATTTTAGTTCCTTCA
381-C-R	TGAAGGAACTAAAATTTTGTCTGGGCTCCGTCAGTCATCTTGCTGCCAAGGGAAGCACA

Supplementary Table 14

DNA methylation analysis primer

Bisulfite Conversion PCR	Sequence (5'-3')
Methy-F	GTGGATTTTGATTAATGGGT
Methy-R	AAAACTTTCCCAAAAATTCC

Supplementary Table 15

Primers used to construct luciferase reporter plasmid

Primer name	
LUC-IRF8-F	TCTGCGATCTAAGTAAGGACCTCTTAACGCTGACG
LUC-IRF8-R	ACCGGAATGCCAAGCTTAGGCAAAAAGCAGAGAGGG
LUC-381-F(NheI)	CTAGCTAGCTACCATAGGAAACAGGCCTGGAG
LUC-381-R(SacI)	CGAGCTCGTGAGAAGTGCCTATGCTGCC
