# Supplementary material for: Lupus enhancer risk variant causes dysregulation of IRF8 through cooperative lncRNA and DNA methylation machinery
Source: Nat Commun. 2022 Apr 6;13:1855. doi: 10.1038/s41467-022-29514-y (PMC8987079; doi:10.1038/s41467-022-29514-y)
Supplement: Supplementary file 3 — Description of Additional Supplementary Files [file 41467_2022_29514_MOESM3_ESM.pdf]

Title: Supplementary Data 1:
Description: Autoimmune disease associated genetic variants in *IRF8* locus with genome-wide significance

Title: Supplementary Data 2:
Description: SNPs in tight LD (r2>0.8) with tag variants

Title: Supplementary Data 3:
Description: Analysis of the enhancer marks of genetic variants in different immune cell subpopulations

Title: Supplementary Data 4:
Description: Differential gene expression list between WT group and KO group

Title: Supplementary Data 5:
Description: The list of genes whose promoter exists interaction with rs2280381 site

Title: Supplementary Data 6:
Description: RNA sequencing analysis of the expression of genes whose promoter exists interaction with rs2280381 site

Title: Supplementary Data 7:
Description: RNA sequencing analysis of the expression of the direct targets of IRF8 predicted by IPA analysis and IRF8 ChIP-Seq in monocytes

Title: Supplementary Data 8:
Description: GO pathway analysis for the differentially expressed genes

Title: Supplementary Data 9:
Description: DAPA-MS experiment and ChIP-seq identified proteins binding to the rs2280381 sequence

Title: Supplementary Data 10:
Description: The genomic regions exist connection with IRF8 promoter detected by 4C-seq
