# Supplementary material for: Lupus enhancer risk variant causes dysregulation of IRF8 through cooperative lncRNA and DNA methylation machinery
Source: Nat Commun. 2022 Apr 6;13:1855. doi: 10.1038/s41467-022-29514-y (PMC8987079; doi:10.1038/s41467-022-29514-y)
Supplement: Supplementary file 14 — Reporting Summary [file 41467_2022_29514_MOESM14_ESM.pdf]

nature portfolio

Corresponding author(s): Nan Shen

Last updated by author(s): Mar 8, 2022

Reporting Summary

Nature Portfolio wishes to improve the reproducibility of the work that we publish. This form provides structure for consistency and transparency in reporting. For further information on Nature Portfolio policies, see our Editorial Policies and the Editorial Policy Checklist.

Statistics

For all statistical analyses, confirm that the following items are present in the figure legend, table legend, main text, or Methods section.

n/a	Confirmed	
☐	☒	The exact sample size (*n*) for each experimental group/condition, given as a discrete number and unit of measurement
☐	☒	A statement on whether measurements were taken from distinct samples or whether the same sample was measured repeatedly
☐	☒	The statistical test(s) used AND whether they are one- or two-sided<br>*Only common tests should be described solely by name; describe more complex techniques in the Methods section.*
☒	☐	A description of all covariates tested
☒	☐	A description of any assumptions or corrections, such as tests of normality and adjustment for multiple comparisons
☐	☒	A full description of the statistical parameters including central tendency (e.g. means) or other basic estimates (e.g. regression coefficient) AND variation (e.g. standard deviation) or associated estimates of uncertainty (e.g. confidence intervals)
☐	☒	For null hypothesis testing, the test statistic (e.g. *F*, *t*, *r*) with confidence intervals, effect sizes, degrees of freedom and *P* value noted<br>*Give P values as exact values whenever suitable.*
☒	☐	For Bayesian analysis, information on the choice of priors and Markov chain Monte Carlo settings
☒	☐	For hierarchical and complex designs, identification of the appropriate level for tests and full reporting of outcomes
☒	☐	Estimates of effect sizes (e.g. Cohen's *d*, Pearson's *r*), indicating how they were calculated

Our web collection on statistics for biologists contains articles on many of the points above.

Software and code

Policy information about availability of computer code

Data collection	QuantStudio Real-Time PCR software (ABI, v1.2), HiSeq Control Software (HCS) (v3.3.76.1), GAPipeline-1.6, OLB (v1.9.3), the genomic sequences were collected from UCSC Genome Browser on Human (GRCh37/hg19)(http://genome.ucsc.edu/cgi-bin/hgGateway) .
Data analysis	GraphPad Prism 8, R version 3.3.3, MARIO (v3.93), Bowtie-align (version 1.2),4Cseqpipe (version 0.7), circus (v0.69-6), Samtools (v0.1.19), HT-seq (v0.11.2), DESeq2 (v1.24.0), cutadapt(v1.15), Hisat2 (v2.1.0), Mascot Daemon (v2.3.0), CHOPCHOP (version 3), ADASTRA(v3.5.2), Cistrome (http://cistrome.org/db/#/, no available version number), pegFinder(http://pegfinder.sidichenlab.org/,no available version number), ImmuNexUT (https://www.immunexut.org/, no available version number), ICE V3.0 (https://ice.synthego.com/#/),NIH Roadmap Epigenomics Mapping Consortium (http://www.roadmapepigenomics.org/, no available version number),GTEx (https://gtexportal.org/home/, no available version number),HaploReg v4.1, RPISeq Version 1.0.

For manuscripts utilizing custom algorithms or software that are central to the research but not yet described in published literature, software must be made available to editors and reviewers. We strongly encourage code deposition in a community repository (e.g. GitHub). See the Nature Portfolio guidelines for submitting code & software for further information.

Data

Policy information about availability of data

All manuscripts must include a data availability statement. This statement should provide the following information, where applicable:

- Accession codes, unique identifiers, or web links for publicly available datasets
- A description of any restrictions on data availability
- For clinical datasets or third party data, please ensure that the statement adheres to our policy

The RNA sequencing data generated in this study have been deposited in the ArrayExpress database at EMBL-EBI under accession code E-MTAB-11306 (https://

www.ebi.ac.uk/arrayexpress/experiments/E-MTAB-11306/).
The 4C sequencing data generated in this study have been deposited in the ArrayExpress database at EMBL-EBI under accession code E-MTAB-10120 (https://www.ebi.ac.uk/arrayexpress/experiments/E-MTAB-10120/) and E-MTAB-11175 (https://www.ebi.ac.uk/arrayexpress/experiments/E-MTAB-11175/).
The mass spectrometry proteomics data generated in this study have been deposited in the PRIDE database under accession code PXD029688 (https:/www.ebi.ac.uk/pride/archive/projects/PXD029688).
The public ATAC sequencing data E-MTAB-8982 (https://www.ebi.ac.uk/arrayexpress/experiments/E-MTAB-8982/) was used to analyze the chromatin accessibility of primary immune cells.
The data from GSE45291 (https://www.ncbi.nlm.nih.gov/geo/query/acc.cgi?acc=GSE45291), GSE108497 (https://www.ncbi.nlm.nih.gov/geo/query/acc.cgi?acc=GSE108497) and GSE110174 (https://www.ncbi.nlm.nih.gov/geo/query/acc.cgi?acc=GSE110174) was used to analyze IRF8 expression in health control and SLE patients.
The data from GSE149050 (https://www.ncbi.nlm.nih.gov/geo/query/acc.cgi?acc=GSE149050) was used to analyze the expression of AC092723.1 in different immune cell subpopulations.
GSE155555 (https://www.ncbi.nlm.nih.gov/geo/query/acc.cgi?acc=GSE155555) data was used to analyze the ATAC-seq and H3K27ac signal of U-937 cells.
The NIH Roadmap Epigenomics Mapping Consortium(http://www.roadmapepigenomics.org/) was used to analyze the enhancer signal of different immune cell subpopulations.
Cistrome database (http://cistrome.org/db/#/) and ADASTRA database (https://adastra.autosome.ru/susan) were used to analyze the binding proteins to rs2280381 in this study.
GTEx (https://gtexportal.org/home/) and ImmuNexUT database (https://www.immunexut.org/) were used to analyze the eQTL result in this study.
All other remaining data are available within the Article and Supplementary Files.
Source data are provided with this paper.

Field-specific reporting

Please select the one below that is the best fit for your research. If you are not sure, read the appropriate sections before making your selection.

[x] Life sciences   [ ] Behavioural & social sciences   [ ] Ecological, evolutionary & environmental sciences

For a reference copy of the document with all sections, see nature.com/documents/nr-reporting-summary-flat.pdf

Life sciences study design

All studies must disclose on these points even when the disclosure is negative.

Sample size	No statistical method was used to predetermine the sample size. Experiments were performed with three biological replicates as reported in the Statistics and figure legends. For clinical sample analysis, the sample size was determined by similar researches(Ban et al. Nat Commun. 2021, PMID:34282144). The sample size in this study had enough power to evaluate the express significance of IRF8 and AC092723.1 in SLE and health subjects.
Data exclusions	No data were excluded
Replication	There were no difficulties with the reproduction of any of the reported findings. All experiments for this study are from multiple biologically independent experiments and biologically independent samples, with "n" and replication numbers reported in the legend of each Figure.
Randomization	Randomization was not relevant to this study as all experiments were performed with molecular/cell biology techniques in cell lines where the experimenter designs and performs the experimental conditions so randomization between conditions is not possible.
Blinding	Investigators were not blinded to group allocation as data analysis involved in all cases objective measurement methods are not affected by investigator bias.

Behavioural & social sciences study design

All studies must disclose on these points even when the disclosure is negative.

Study description	*Briefly describe the study type including whether data are quantitative, qualitative, or mixed-methods (e.g. qualitative cross-sectional, quantitative experimental, mixed-methods case study).*
Research sample	*State the research sample (e.g. Harvard university undergraduates, villagers in rural India) and provide relevant demographic information (e.g. age, sex) and indicate whether the sample is representative. Provide a rationale for the study sample chosen. For studies involving existing datasets, please describe the dataset and source.*
Sampling strategy	*Describe the sampling procedure (e.g. random, snowball, stratified, convenience). Describe the statistical methods that were used to predetermine sample size OR if no sample-size calculation was performed, describe how sample sizes were chosen and provide a rationale for why these sample sizes are sufficient. For qualitative data, please indicate whether data saturation was considered, and what criteria were used to decide that no further sampling was needed.*
Data collection	*Provide details about the data collection procedure, including the instruments or devices used to record the data (e.g. pen and paper, computer, eye tracker, video or audio equipment) whether anyone was present besides the participant(s) and the researcher, and whether the researcher was blind to experimental condition and/or the study hypothesis during data collection.*

Timing	*Indicate the start and stop dates of data collection. If there is a gap between collection periods, state the dates for each sample cohort.*
Data exclusions	*If no data were excluded from the analyses, state so OR if data were excluded, provide the exact number of exclusions and the rationale behind them, indicating whether exclusion criteria were pre-established.*
Non-participation	*State how many participants dropped out/declined participation and the reason(s) given OR provide response rate OR state that no participants dropped out/declined participation.*
Randomization	*If participants were not allocated into experimental groups, state so OR describe how participants were allocated to groups, and if allocation was not random, describe how covariates were controlled.*

Ecological, evolutionary & environmental sciences study design

All studies must disclose on these points even when the disclosure is negative.

Study description	*Briefly describe the study. For quantitative data include treatment factors and interactions, design structure (e.g. factorial, nested, hierarchical), nature and number of experimental units and replicates.*
Research sample	*Describe the research sample (e.g. a group of tagged Passer domesticus, all Stenocereus thurberi within Organ Pipe Cactus National Monument), and provide a rationale for the sample choice. When relevant, describe the organism taxa, source, sex, age range and any manipulations. State what population the sample is meant to represent when applicable. For studies involving existing datasets, describe the data and its source.*
Sampling strategy	*Note the sampling procedure. Describe the statistical methods that were used to predetermine sample size OR if no sample-size calculation was performed, describe how sample sizes were chosen and provide a rationale for why these sample sizes are sufficient.*
Data collection	*Describe the data collection procedure, including who recorded the data and how.*
Timing and spatial scale	*Indicate the start and stop dates of data collection, noting the frequency and periodicity of sampling and providing a rationale for these choices. If there is a gap between collection periods, state the dates for each sample cohort. Specify the spatial scale from which the data are taken*
Data exclusions	*If no data were excluded from the analyses, state so OR if data were excluded, describe the exclusions and the rationale behind them, indicating whether exclusion criteria were pre-established.*
Reproducibility	*Describe the measures taken to verify the reproducibility of experimental findings. For each experiment, note whether any attempts to repeat the experiment failed OR state that all attempts to repeat the experiment were successful.*
Randomization	*Describe how samples/organisms/participants were allocated into groups. If allocation was not random, describe how covariates were controlled. If this is not relevant to your study, explain why.*
Blinding	*Describe the extent of blinding used during data acquisition and analysis. If blinding was not possible, describe why OR explain why blinding was not relevant to your study.*

Did the study involve field work? ☐ Yes ☐ No

Field work, collection and transport

Field conditions	*Describe the study conditions for field work, providing relevant parameters (e.g. temperature, rainfall).*
Location	*State the location of the sampling or experiment, providing relevant parameters (e.g. latitude and longitude, elevation, water depth).*
Access & import/export	*Describe the efforts you have made to access habitats and to collect and import/export your samples in a responsible manner and in compliance with local, national and international laws, noting any permits that were obtained (give the name of the issuing authority, the date of issue, and any identifying information).*
Disturbance	*Describe any disturbance caused by the study and how it was minimized.*

Reporting for specific materials, systems and methods

We require information from authors about some types of materials, experimental systems and methods used in many studies. Here, indicate whether each material, system or method listed is relevant to your study. If you are not sure if a list item applies to your research, read the appropriate section before selecting a response.

Materials & experimental systems

n/a	Involved in the study
☐	☒ Antibodies
☐	☒ Eukaryotic cell lines
☒	☐ Palaeontology and archaeology
☒	☐ Animals and other organisms
☐	☒ Human research participants
☒	☐ Clinical data
☒	☐ Dual use research of concern

Methods

n/a	Involved in the study
☒	☐ ChIP-seq
☒	☐ Flow cytometry
☒	☐ MRI-based neuroimaging

Antibodies

Antibodies used

Anti-H3K27ac (# ab177178, Abcam, 2 μg for 25 μg of chromatin). Anti-IRF8(#5628S,Cell Signaling Technology,1:1000 for western blot). Anti-TET1(#61741, Active motif, 2μg per 10^7 cell lysate in RIP assay and 4 μl per ChIP assay). Anti-KAT3B/p300 (#ab10485,Abcam, 2μg per 10^7 cell lysate). Anti-WDR5(#ab56919,Abcam, 2μg per 10^7 cell lysate). Anti-DOT1L (#A300-953A,Thermo, 2μg per 10^7 cell lysate). anti-PU.1 (#2266S, Cell Signaling Technology, 1:50 for one ChIP sample),HRP-conjugated GAPDH Mouse mAb(#AC035,ABclonal,1:5000 for western blot) .

Validation

Anti-Histone H3 (acetyl K27) Rabbit mAb(# ab177178, Abcam, 2 μg for 25 μg of chromatin) have been cited in 35 publications, Application: Flow Cyt, ICC, PepArr, IHC-P, WB, ChIP, ChIP-seq. Species reactivity: Mouse, Rat, Human. please see https://www.abcam.cn/histone-h3-acetyl-k27-antibody-ep16602-chip-grade-ab177178.html.
Anti-IRF8 Rabbit mAb(#5628S,Cell Signaling Technology,1:1000 for western blot) has been cited in 31 publications, Application:WB, IP, ChIP, ChIP-seq, IF. Species Reactivity:Human, Mouse. please see https://www.cellsignal.cn/products/primary-antibodies/irf-8-d20d8-rabbit-mab/5628?site-search-type=Products&N=4294956287&Ntt=5628s&fromPage=plp&_requestid=287621.
HRP-conjugated GAPDH Mouse mAb(#AC035,ABclonal,1:5000 for western blot) has been cited in 6 publications, Application:WB. Species Reactivity: Human, Mouse, Rat. please see https://abclonal.com.cn/catalog/AC035.
Anti-Tet1 antibody (mAb) (#61741, Active motif, 2μg per 10^7 cell lysate in RIP assay and 4 μl per ChIP assay). Application: ChIP-qPCR, IHC(F)/IF WB. Species Reactivity:Mouse, Human. please see https://www.activemotif.com/catalog/details/61741/tet1-antibody-mab-clone-5d6.
Anti-KAT3B/p300 Rabbit mAb(#ab10485,Abcam, 2μg for 1x10^7 cell lyaste) has been cited in 25 publications, Application:IP, WB. Species Reactivity:Human. please see https://www.abcam.cn/kat3b--p300-antibody-ab10485.html.
Anti-WDR5 Mouse mAb(#ab56919,Abcam, 2μg for 1x10^7 cell lyaste) has been cited in 29 publications, Application: WB, ICC/IF, ChIP, Flow Cyt. Species Reactivity:Human. please see https://www.abcam.cn/wdr5-antibody-2c2-ab56919.html.
Anti-DOT1L Rabbit pAb (#A300-953A,Thermo, 2μg for 1x10^7 cell lyaste),Application:WB,IHC,IP,ChIP-seq. Species Reactivity:Human. please see https://www.thermofisher.cn/cn/zh/antibody/product/DOT1L-Antibody-Polyclonal/A300-953A.
Anti-PU.1 Rabbit mAb (#2266S, Cell Signaling Technology, 1:50 for one ChIP sample) have been cited in 44 publications, Application:WB, IP,IHC,IF,F,ChIP.Species Reactivity:Human. please see https://www.cellsignal.cn/products/primary-antibodies/pu-1-antibody/2266?site-search-type=Products&N=4294956287&Ntt=2266s&fromPage=plp&_requestid=289035

Eukaryotic cell lines

Policy information about cell lines

Cell line source(s)

U-937, Raji, Jurkat and HEK293T. All cell lines were obtained from Cell Bank of Chinese Academy of Science (Shanghai, China).

Authentication

All cell lines authenticated by STR method.

Mycoplasma contamination

All cell lines were tested negative for mycoplasma contamination.

Commonly misidentified lines (See ICLAC register)

No commonly misidentified cell lines were used.

Palaeontology and Archaeology

Specimen provenance

Provide provenance information for specimens and describe permits that were obtained for the work (including the name of the issuing authority, the date of issue, and any identifying information). Permits should encompass collection and, where applicable, export.

Specimen deposition

Indicate where the specimens have been deposited to permit free access by other researchers.

Dating methods

If new dates are provided, describe how they were obtained (e.g. collection, storage, sample pretreatment and measurement), where they were obtained (i.e. lab name), the calibration program and the protocol for quality assurance OR state that no new dates are provided.

☐ Tick this box to confirm that the raw and calibrated dates are available in the paper or in Supplementary Information.

Ethics oversight	*Identify the organization(s) that approved or provided guidance on the study protocol, OR state that no ethical approval or guidance was required and explain why not.*

Note that full information on the approval of the study protocol must also be provided in the manuscript.

Animals and other organisms

Policy information about studies involving animals; ARRIVE guidelines recommended for reporting animal research

Laboratory animals	*For laboratory animals, report species, strain, sex and age OR state that the study did not involve laboratory animals.*
Wild animals	*Provide details on animals observed in or captured in the field; report species, sex and age where possible. Describe how animals were caught and transported and what happened to captive animals after the study (if killed, explain why and describe method; if released, say where and when) OR state that the study did not involve wild animals.*
Field-collected samples	*For laboratory work with field-collected samples, describe all relevant parameters such as housing, maintenance, temperature, photoperiod and end-of-experiment protocol OR state that the study did not involve samples collected from the field.*
Ethics oversight	*Identify the organization(s) that approved or provided guidance on the study protocol, OR state that no ethical approval or guidance was required and explain why not.*

Note that full information on the approval of the study protocol must also be provided in the manuscript.

Human research participants

Policy information about studies involving human research participants

Population characteristics	IRF8 and AC092723.1 expression analysis: RNA Samples from SLE patients (as defined using the ACR classification criteria for lupus – Hochberg MC Arthritis Rheum 40:1725, ACR-1997 criteria) (32 SLE patients, all female, the age range from 27-63 and the average age is 41.90) and healthy control subjects (26 health subjects, all female, the age range from 22-58 and the average age is 39.80) were extracted to detect the expression of IRF8 and AC092723.1.<br><br>For PBMCs used in vitro editing, Health participants were 1 female and 2 males (age of 25, 26, 32 years), and blood samples were collected for in vitro editing.
Recruitment	Individuals were recruited by the internal review and ethics boards of Renji Hospital, Shanghai Jiao Tong University School of Medicine (SJTUSM) and signed informed consent. SLE patients were diagnosed based on the ACR-1997 criteria, with no cyclophosphamide or glucocorticoid shock therapy in the latest one month. Healthy donors were recruited with age and gender-matched with SLE patients, no chronic diseases, and acute infection.
Ethics oversight	All samples were collected with signed informed consent according to the internal review and ethics boards of Renji Hospital, Shanghai Jiao Tong University. The experiments were approved by the internal review and ethics boards of Renji Hospital, Shanghai Jiao Tong University School of Medicine (SJTUSM).

Note that full information on the approval of the study protocol must also be provided in the manuscript.

Clinical data

Policy information about clinical studies

All manuscripts should comply with the ICMJEguidelines for publication of clinical research and a completedCONSORT checklist must be included with all submissions.

Clinical trial registration	*Provide the trial registration number from ClinicalTrials.gov or an equivalent agency.*
Study protocol	*Note where the full trial protocol can be accessed OR if not available, explain why.*
Data collection	*Describe the settings and locales of data collection, noting the time periods of recruitment and data collection.*
Outcomes	*Describe how you pre-defined primary and secondary outcome measures and how you assessed these measures.*

Dual use research of concern

Policy information about dual use research of concern

Hazards

Could the accidental, deliberate or reckless misuse of agents or technologies generated in the work, or the application of information presented in the manuscript, pose a threat to:

No	Yes	
☐	☐	Public health
☐	☐	National security
☐	☐	Crops and/or livestock
☐	☐	Ecosystems
☐	☐	Any other significant area

Experiments of concern

Does the work involve any of these experiments of concern:

No	Yes	
☐	☐	Demonstrate how to render a vaccine ineffective
☐	☐	Confer resistance to therapeutically useful antibiotics or antiviral agents
☐	☐	Enhance the virulence of a pathogen or render a nonpathogen virulent
☐	☐	Increase transmissibility of a pathogen
☐	☐	Alter the host range of a pathogen
☐	☐	Enable evasion of diagnostic/detection modalities
☐	☐	Enable the weaponization of a biological agent or toxin
☐	☐	Any other potentially harmful combination of experiments and agents

ChIP-seq

Data deposition

☐ Confirm that both raw and final processed data have been deposited in a public database such as GEO.

☐ Confirm that you have deposited or provided access to graph files (e.g. BED files) for the called peaks.

Data access links<br>*May remain private before publication.*	*For "Initial submission" or "Revised version" documents, provide reviewer access links. For your "Final submission" document, provide a link to the deposited data.*
Files in database submission	*Provide a list of all files available in the database submission.*
Genome browser session<br>(e.g. UCSC)	*Provide a link to an anonymized genome browser session for "Initial submission" and "Revised version" documents only, to enable peer review. Write "no longer applicable" for "Final submission" documents.*

Methodology

Replicates	*Describe the experimental replicates, specifying number, type and replicate agreement.*
Sequencing depth	*Describe the sequencing depth for each experiment, providing the total number of reads, uniquely mapped reads, length of reads and whether they were paired- or single-end.*
Antibodies	*Describe the antibodies used for the ChIP-seq experiments; as applicable, provide supplier name, catalog number, clone name, and lot number.*
Peak calling parameters	*Specify the command line program and parameters used for read mapping and peak calling, including the ChIP, control and index files used.*
Data quality	*Describe the methods used to ensure data quality in full detail, including how many peaks are at FDR 5% and above 5-fold enrichment.*
Software	*Describe the software used to collect and analyze the ChIP-seq data. For custom code that has been deposited into a community repository, provide accession details.*

Flow Cytometry

Plots

Confirm that:

- [ ] The axis labels state the marker and fluorochrome used (e.g. CD4-FITC).
- [ ] The axis scales are clearly visible. Include numbers along axes only for bottom left plot of group (a 'group' is an analysis of identical markers).
- [ ] All plots are contour plots with outliers or pseudocolor plots.
- [ ] A numerical value for number of cells or percentage (with statistics) is provided.

Methodology

Sample preparation	*Describe the sample preparation, detailing the biological source of the cells and any tissue processing steps used.*
Instrument	*Identify the instrument used for data collection, specifying make and model number.*
Software	*Describe the software used to collect and analyze the flow cytometry data. For custom code that has been deposited into a community repository, provide accession details.*
Cell population abundance	*Describe the abundance of the relevant cell populations within post-sort fractions, providing details on the purity of the samples and how it was determined.*
Gating strategy	*Describe the gating strategy used for all relevant experiments, specifying the preliminary FSC/SSC gates of the starting cell population, indicating where boundaries between "positive" and "negative" staining cell populations are defined.*

- [ ] Tick this box to confirm that a figure exemplifying the gating strategy is provided in the Supplementary Information.

Magnetic resonance imaging

Experimental design

Design type	*Indicate task or resting state; event-related or block design.*
Design specifications	*Specify the number of blocks, trials or experimental units per session and/or subject, and specify the length of each trial or block (if trials are blocked) and interval between trials.*
Behavioral performance measures	*State number and/or type of variables recorded (e.g. correct button press, response time) and what statistics were used to establish that the subjects were performing the task as expected (e.g. mean, range, and/or standard deviation across subjects).*

Acquisition

Imaging type(s)	*Specify: functional, structural, diffusion, perfusion.*
Field strength	*Specify in Tesla*
Sequence & imaging parameters	*Specify the pulse sequence type (gradient echo, spin echo, etc.), imaging type (EPI, spiral, etc.), field of view, matrix size, slice thickness, orientation and TE/TR/flip angle.*
Area of acquisition	*State whether a whole brain scan was used OR define the area of acquisition, describing how the region was determined.*

Diffusion MRI ☐ Used ☐ Not used

Preprocessing

Preprocessing software	*Provide detail on software version and revision number and on specific parameters (model/functions, brain extraction, segmentation, smoothing kernel size, etc.).*
Normalization	*If data were normalized/standardized, describe the approach(es): specify linear or non-linear and define image types used for transformation OR indicate that data were not normalized and explain rationale for lack of normalization.*
Normalization template	*Describe the template used for normalization/transformation, specifying subject space or group standardized space (e.g. original Talairach, MNI305, ICBM152) OR indicate that the data were not normalized.*
Noise and artifact removal	*Describe your procedure(s) for artifact and structured noise removal, specifying motion parameters, tissue signals and physiological signals (heart rate, respiration).*

Volume censoring | *Define your software and/or method and criteria for volume censoring, and state the extent of such censoring.*

Statistical modeling & inference

Model type and settings | *Specify type (mass univariate, multivariate, RSA, predictive, etc.) and describe essential details of the model at the first and second levels (e.g. fixed, random or mixed effects; drift or auto-correlation).*

Effect(s) tested | *Define precise effect in terms of the task or stimulus conditions instead of psychological concepts and indicate whether ANOVA or factorial designs were used.*

Specify type of analysis: ☐ Whole brain ☐ ROI-based ☐ Both

Statistic type for inference
(See Eklund et al. 2016) | *Specify voxel-wise or cluster-wise and report all relevant parameters for cluster-wise methods.*

Correction | *Describe the type of correction and how it is obtained for multiple comparisons (e.g. FWE, FDR, permutation or Monte Carlo).*

Models & analysis

n/a	Involved in the study
☐	☐ Functional and/or effective connectivity
☐	☐ Graph analysis
☐	☐ Multivariate modeling or predictive analysis

Functional and/or effective connectivity | *Report the measures of dependence used and the model details (e.g. Pearson correlation, partial correlation, mutual information).*

Graph analysis | *Report the dependent variable and connectivity measure, specifying weighted graph or binarized graph, subject- or group-level, and the global and/or node summaries used (e.g. clustering coefficient, efficiency, etc.).*

Multivariate modeling and predictive analysis | *Specify independent variables, features extraction and dimension reduction, model, training and evaluation metrics.*
